# Estrogen deficiency impedes fracture healing despite eliminating the excessive absorption of the posterior callus in a semi-fixed distal tibial fracture mouse model

**DOI:** 10.1186/s12891-023-06929-2

**Published:** 2023-10-10

**Authors:** Yunpeng Hu, Qiangqiang Lian, Fuyuan Cao, Xiaoli Hou, Hetong Li, Lei Xing, Mengqin Wang, Faming Tian, Liu Zhang

**Affiliations:** 1 Department of Orthopedic Surgery, Hebei Medical University, Shijiazhuang, Hebei P. R. China; 2 School of Public Health, North China University of Science and Technology, Tangshan, Hebei P. R. China; 3 Emergency Department, JST, The Fourth Clinical Hospital of Peking University, Beijing, P. R. China; 4 Department of Orthopedic Surgery, Emergency General Hospital, Xibahenanli29, Chaoyang Dis, Beijing, 100028 P. R. China

**Keywords:** Distal tibia, Fracture healing, Mouse model, Osteoporosis

## Abstract

**Background:**

Treatment of distal tibial fractures is a challenge due to their specific anatomical location. However, there is no appropriate mouse model to simulate a clinical distal tibial fracture for basic research. The aim of this investigation was to evaluate the feasibility of simulating a clinical fracture of the distal tibia of mice and to investigate the effect of ovariectomy (OVX)-induced osteoporosis on fracture healing in this model.

**Methods:**

Sixty female 8-week-old C57BL/6 mice were randomly divided into two groups, either sham or OVX. A semi-fixation distal tibia fracture was established in the right tibia after 8 weeks of OVX. The right tibias were collected at 7, 14, 21, and 28 days post fracture.

**Results:**

In the semi-fixation distal tibia fracture model, the posterior callus in the sham group showed excessive bone resorption and lower bone mass phenotype compared with the anterior site; a similar trend was not found in the OVX group. At 28 days post fracture, the posterior callus was more mineralized than the anterior callus in the OVX group. Although the fracture healing of the sham group showed a special phenotype in this mode, the progress and quality of fracture healing were still better than those of the OVX group.

**Conclusion:**

A semi-fixed distal tibial closed fracture mouse model was successfully established. In this model, excess bone resorption of the posterior callus impaired normal fracture healing, but not in OVX-induced osteoporotic bone. Although the stress shielding effect was not observed in the OVX group, impaired bone healing caused by OVX was still present. Our results suggest that this fracture model may have potential for studies on distal tibial fractures and stress shielding.

## Background

One of the most common injuries to the musculoskeletal system is bone fracture [1]. In the US alone, roughly 8 million people break a bone every year [2]. Fractures of the tibia, particularly the distal tibia, are at a higher risk of delayed healing and non-union than other long bones due to their special structure and poor blood supply [3, 4]. Distal tibial fractures account for approximately 15% of all tibial fractures [5] and represent a potentially long-term disability that can lead to multiple socio-economic problems [6]. Treatment of tibial fractures is a challenge regardless of joint involvement, and 20–50% of patients are affected by post-operative complications of distal tibial fractures [7].

Estrogen deficiency is one of the causes of aging-related osteoporosis. It has been shown that post-menopausal women have significant bone loss and decreased bone strength in the distal tibia, which undoubtedly increases the risk of fracture [8]. In addition, a series of clinical and animal studies have shown that post-menopause results in reduced angiogenesis and a disturbed immune response, which further increases the difficulty of fracture healing in this area [9]. Previous studies investigating the correlation between stress and OVX fracture healing have primarily utilized extracorporeal stress loading techniques, such as whole-body vibration treatment [10, 11]. However, there is a dearth of research on a straightforward fracture model that can accurately mimic physiological loading changes.

Mouse models amenable to genetic manipulation offer an opportunity to elucidate mechanisms of fracture healing. There is a greater recovery speed in small animals such as rodents than in large animals such as humans, but the fundamental mechanisms are the same [12]. However, there is no appropriate mouse model to simulate a clinical distal tibial fracture. Consequently, most studies use large mammals such as dogs to investigate the stress shielding effect, which increases the cost of the study and reduces its applicability [13]. Our study evaluated the feasibility of establishing a fracture model in the distal tibia in mice to simulate a clinical fracture in humans, investigated the effect of ovariectomy (OVX)-induced osteoporosis on fracture healing in this model, and showed more possibilities for mouse models. This model and the effect of OVX on fracture healing were evaluated by histology, immunohistochemistry, and imaging at 7, 14, 21, and 28 days post fracture.

## Methods

### Mice and treatment

**Fig. 1:**
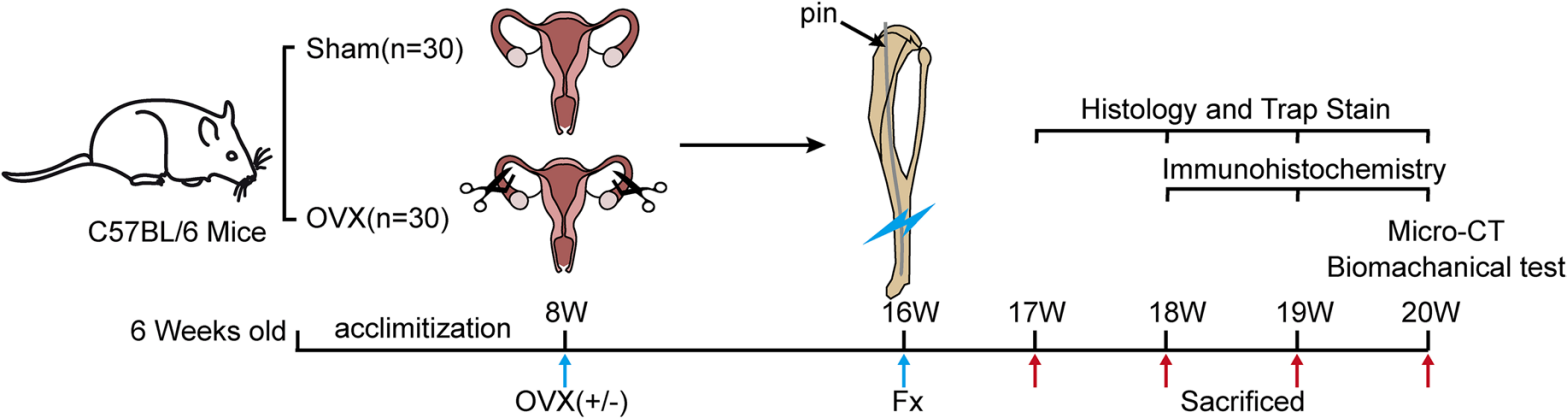
Experimental protocol. OVX: ovariectomized. Fx: fracture

**Fig. 2:**
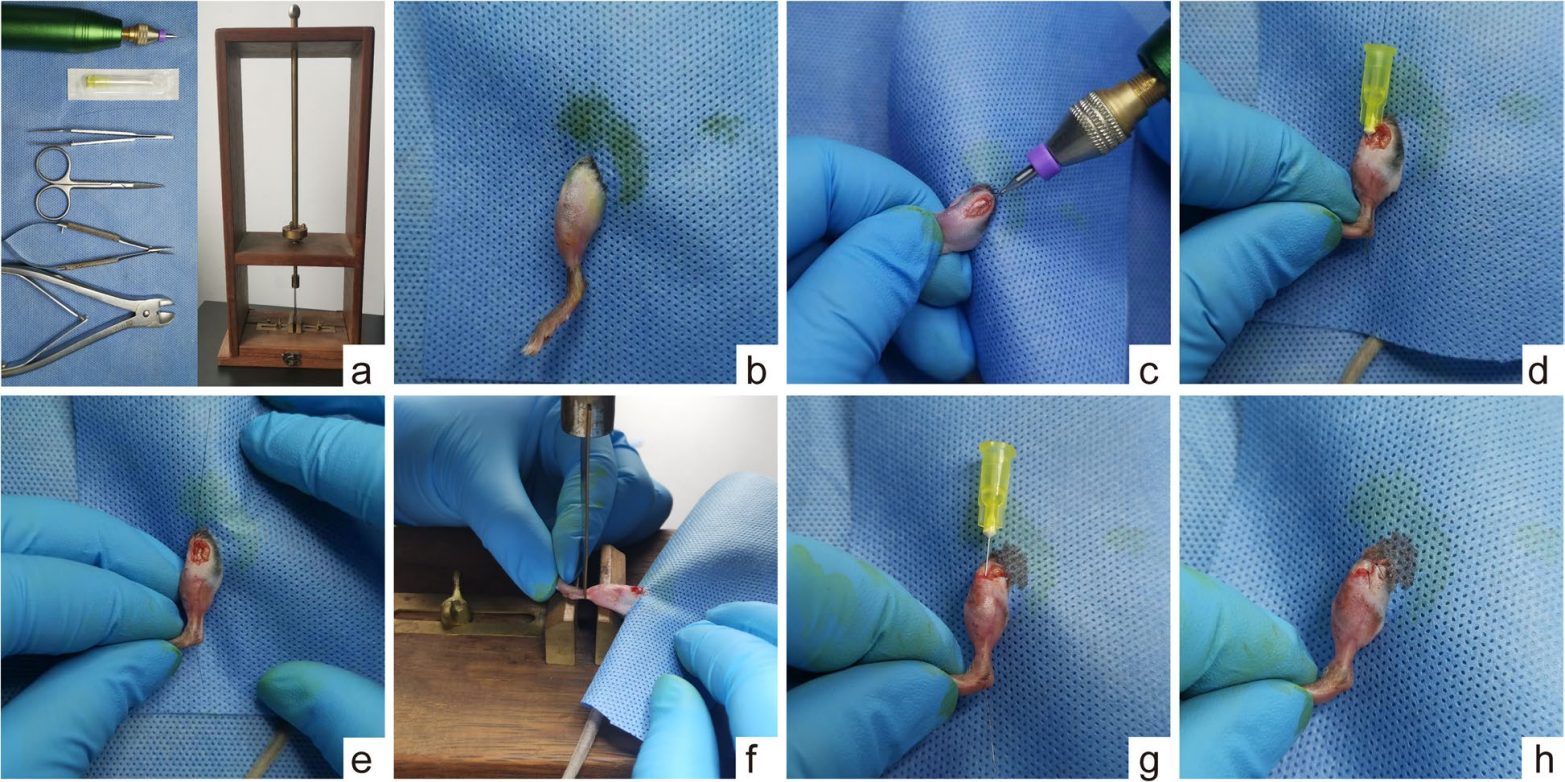
Equipment and Procedure of Distal Tibia Fracture Model. **a** Surgical instruments and homemade three-point bending devices. **b** Skin preparation and sterilization of the surgical area. **c** Drilling on the front of the tibial plateau using a low-speed drill. **d** A needle with a guidewire is passed through the entire length of the tibia from the drilled hole. **e** Withdrawal of the needle with the guidewire remaining. **f** Fracture modelling using a homemade three-point bending device. **g** Reinsertion of the needle along the guidewire. **h** Removal of the guidewire and the excess of the needle, followed by incision closure

Sixty female 6-week-old C57BL/6 mice (Vital River Experimental Animal Technical Co., Ltd., Beijing, China) were randomly divided into two groups: control (sham treatment, *n* = 30) and OVX (*n* = 30) (Fig. 1). All mice were housed with unlimited food and water in an SPF facility with a temperature of 22 ± 2 °C, 50 ± 10% humidity and a 12-h light/dark cycle. After two weeks of acclimatization, these two groups of mice were anesthetized with isoflurane (2% in oxygen) and subjected to either bilateral OVX or sham operation (removal of only the small part of fat tissue around the bilateral ovaries). Eight weeks later, a distal tibia fracture was induced in these now 16-week-old mice. After anesthetization with isoflurane (2% in oxygen), the mice were placed in the supine position and denuded on their right leg, in which was made a 5 mm incision from the medial thigh to the knee. After drilling, the front of the tibial plateau was punctured using a 30 G needle within a 0.1 mm diameter sterile guide wire. The needles were then withdrawn, leaving the guide wire in the bone cavity. Fractures were produced using a custom three-bending device. After confirming the fracture manually, the needle was re-inserted along the guide wire, followed by removal of the extra part of the pin as well as the guide wire, and finally closure of the incision with surgical sutures. Standardized fracture was confirmed by X-ray [14, 15] (Fig. 2). Mice were given buprenorphine (0.1 mg/kg) for 3 days after fracture for pain relief. Six mice in each group were sacrificed with CO_2_ at 7, 14, and 21 d post fracture. The remaining mice were sacrificed at 28 d post fracture (the extra 8 mice in each group at 28 d post fracture were used to undergo three-point bending test, see “Three-point bending test” section). All experiments were approved by the Institutional Animal Care and Use Committee of North China University of Science and Technology (LAEC-NCST-2020204). All methods were carried out in accordance with relevant guidelines and regulations and reported in accordance with ARRIVE guidelines.

### Histological analysis

Fixed samples were then decalcified with 10% EDTA (pH 7.2–7.4) at 4 ℃ twice a week for more than 24 cycles, after which they were able to be easily punctured by 1 mL needle. Samples were then embedded in paraffin, and 5 μm thick sagittal sections were made. Sections were histologically stained after deparaffinization with xylene and gradient alcohol hydration to water. The Modified Saffron-O and Fast Green Staining Kit (G1371, Solarbio, Beijing, China) was used to quantify the bony callus content [16]. All images were blindly quantified and scored by three pathologists.

### Micro-CT analysis

**Fig. 3:**
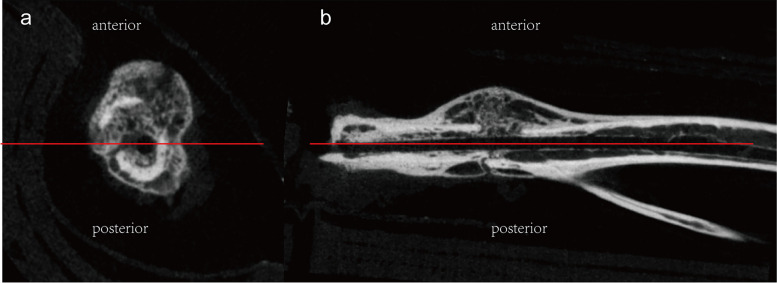
Position of anterior and posterior callus. **a** Cross plane. **b** Sagittal plane

After removing the intramedullary pin, whole right tibias with callus were dissected from the attached muscle and stored in 10% neutral formalin for 48 h. Samples of 28 d post fracture were scanned using a high-resolution micro-CT (SkyScan1176 Software: Version1.1 (build 6), Bruker, Kontich, Belgium) [17]. The region of interest of each callus was the area 2 mm above and below the fracture line [18]. To better describe our findings, we used the coronal plane through the intramedullary pin to divide the callus into anterior and posterior parts (Fig. 3) and calculated architectural parameters in the anterior site, posterior site, and total callus respectively, as previously described [16].

### TRAP staining

The same steps (see “Histological analysis” section) were taken to process the slices, and then Tartrate-resistant acid phosphatase (TRAP) staining was performed to evaluate the density of osteoclasts in the callus area following a standard protocol (G1492, Solarbio, Beijing, China). Measurement processes were performed as previously described [19].

### Immunohistochemistry

The expression of type 1 collagen (Col-I) in each callus was quantified by examining bone sections prepared for histological analysis as previously described [17]. Briefly, sections were deparaffinized, rehydrated, and repaired by 0.05% trypsin at 37 °C for 30 min. Incubation with anti-Col-I (1:200, BA0325; Boster Bio) was performed at 4 °C for 10 h. DAB Kit (ZSGB-BIO Corporation, Beijing, China) was used to develop positive expression. The assessment area was the external callus near the fracture line. The mean intensity of optical density was measured as previously described [20].

### Three-point bending test

For biomechanical analysis, the right tibias with callus were resected at 28 d post fracture (*n* = 6 for each group) and detached from the soft tissue. The mechanical properties of the callus were evaluated immediately after harvest using a universal testing machine (MMT-250NV-10; Shimadzu, Kyoto, Japan). The tibial callus (the anterior surface facing up) was placed on the middle of the two support bars (6 mm) and subjected to a downward force with a speed of 1.0 mm/min until mechanical failure of the callus occurred [21]. Parameters including maximum loading (N) and energy to failure (mJ) were calculated.

### Data analysis and statistics

All data are presented as mean ± SD. Statistical differences were calculated using Student's *t* test. The level of statistical significance was accepted at *P* values < 0.05. Statistical analysis data were measured using SPSS software (SPSS v20.0; IBM, Armonk, New York, USA).

## Results

### Histology

**Fig. 4:**
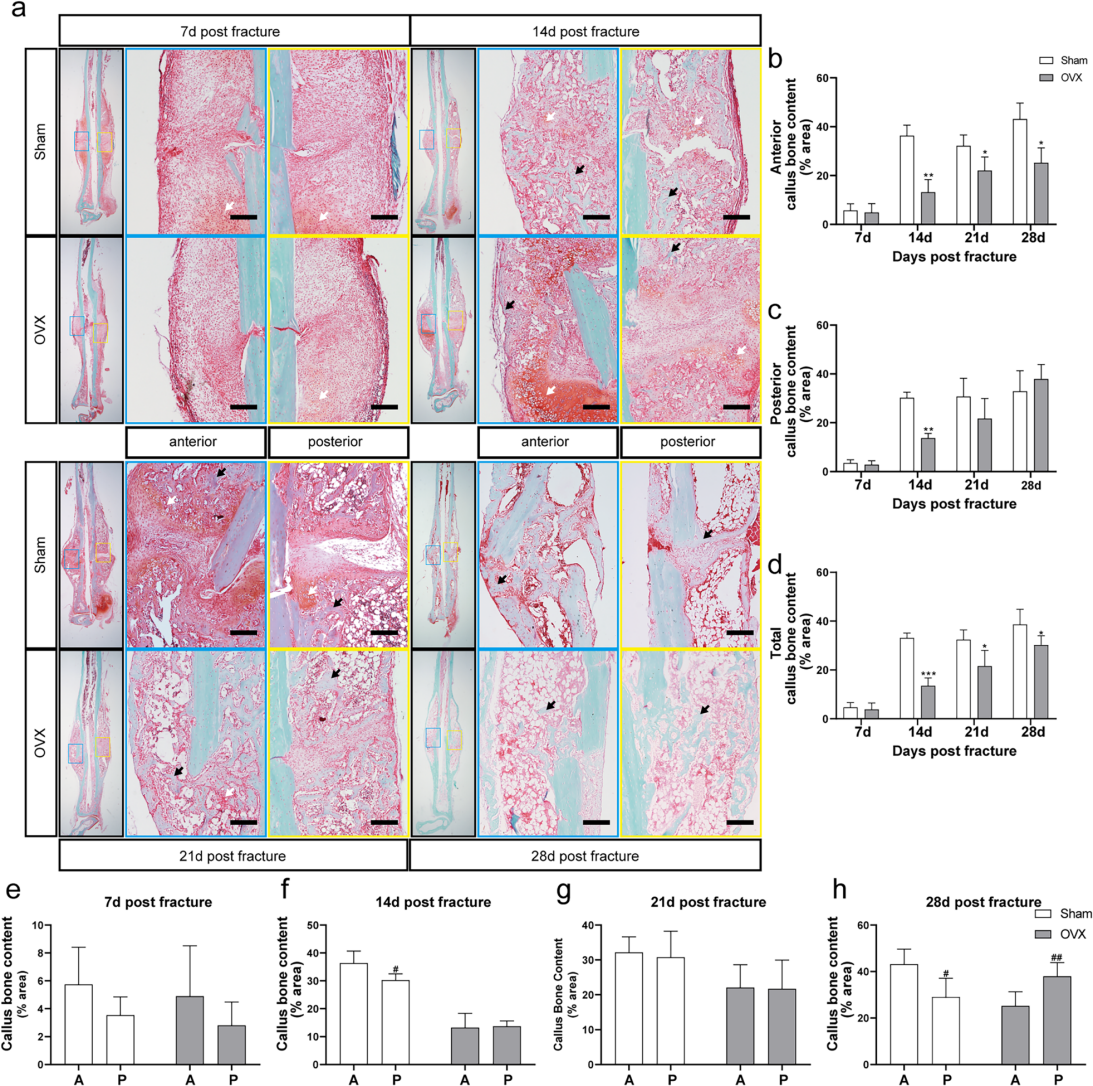
Histology of callus in different time points. **a** Histology demonstrating the sagittal sections of callus from 7, 14, 21, and 28 d post fracture. The black arrow presents bony callus. The white arrow presents cartilage callus. Bar = 200 µm. **b-d** Bone content of anterior, posterior, and total callus at different time points. **e–h** Comparison of bone content between anterior and posterior callus at different time points. A: anterior callus. P: posterior callus. Data are presented as mean ± SD. ^*^P < 0.05 *vs.* sham group; ^**^P < 0.01 *vs.* sham group; ^***^P < 0.001 *vs.* sham group; ^#^*P* < 0.05 *vs.* anterior callus; ^##^*P* < 0.01 *vs.* anterior callus; ^###^*P* < 0.001 *vs.* anterior callus

Histological observations of the callus of the two groups at each of the four time points are presented in Fig. 4a. Bony callus content was significantly reduced in the OVX group at 14, 21, and 28 d post fracture compared with sham (*P* < 0.05 for each). In the posterior callus, the OVX group was significantly lower than the sham group only at 14 d post fracture (*P* < 0.05; Fig. 4b-d). Bony callus content in the posterior side of the sham group was significantly lower than that in the anterior site at 14 and 28 d post fracture (*P* < 0.05 for each; Fig. 4e-h). In contrast, the bony callus content was significantly higher in the posterior site than in the anterior site in the OVX group at 28 d post fracture (*P* < 0.05).

### Micro-CT

**Fig. 5:**
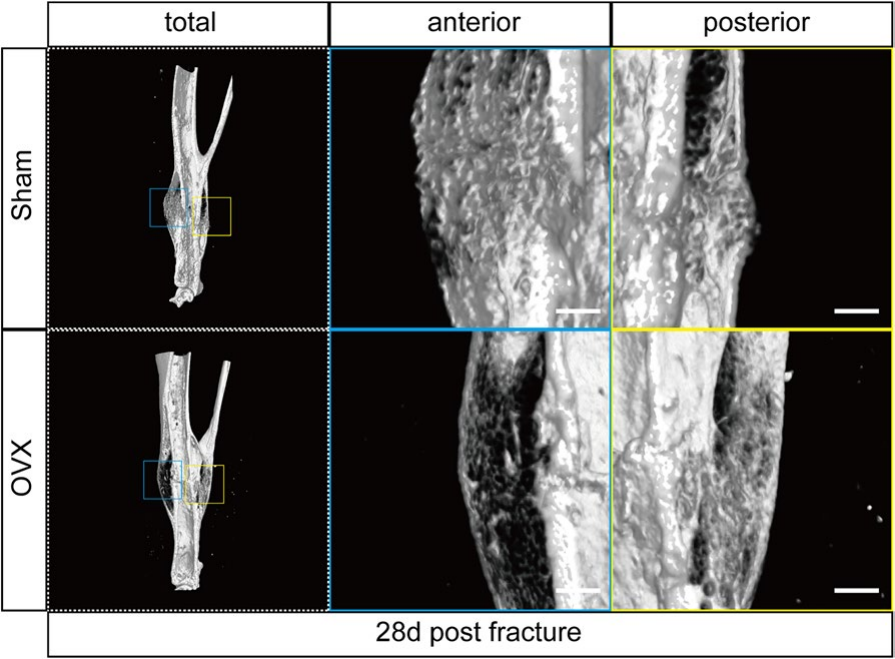
Micro-CT 3D reconstruction images at 28 d post fracture. Bar = 500 µm

**Fig. 6:**
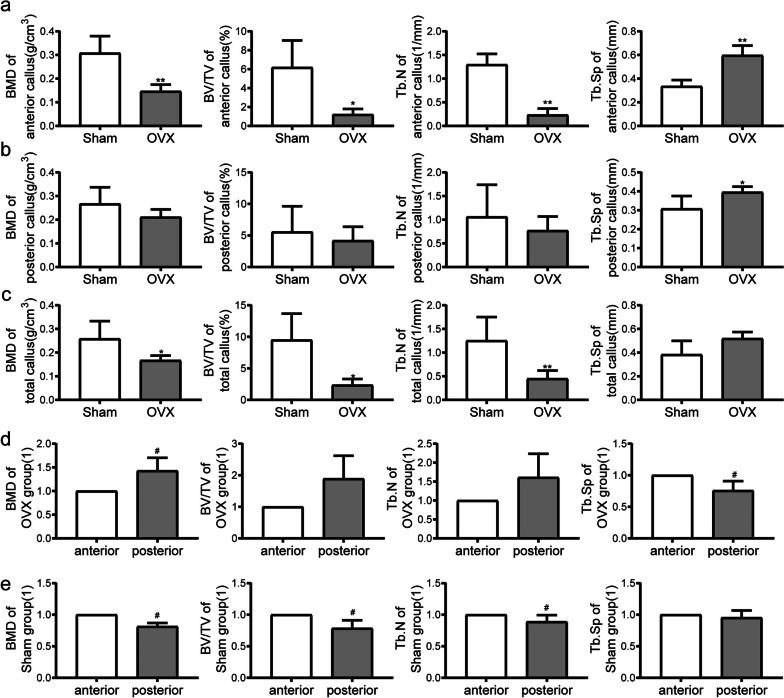
Micro-CT analysis at 28 d post fracture. **a-c** Bone mineral density (BMD), bone volume/total volume (BV/TV), trabecular number (Tb.N), and trabecular separation (Tb.Sp) in anterior, posterior, and total calluses. **d-e** Comparison of BMD, BV/TV, Tb.N, and Tb.Sp between anterior and posterior callus in sham and OVX group. Data are presented as the mean ± SD. ^*^P < 0.05 *vs.* sham group; ^**^P < 0.01 *vs.* sham group; ^***^P < 0.001 *vs.* sham group, ^#^*P* < 0.05 *vs*. anterior callus; ^##^*P* < 0.01 *vs.* anterior callus; ^###^*P* < 0.001 *vs.* anterior callus

3D-reconstructed micro-CT images are shown in Fig. 5. Microstructure parameters of the anterior, posterior, and total callus were quantified separately for 28 d post fracture samples (Fig. 6a-c). In both anterior and total calluses, bone mineral density (BMD), bone volume/total volume (BV/TV), and trabecular number (Tb.N) were significantly decreased, and trabecular separation (Tb.Sp) was significantly increased in the OVX group compared with the sham group (all *P* < 0.05). In the posterior callus, Tb.Sp was significantly increased in the OVX group (*P* < 0.05 *vs.* sham group). Comparing the posterior to the anterior callus, in the sham group, BMD decreased by 18.69%, BV/TV decreased by 21.52%, and the Tb.N decreased by 12.34% in the posterior callus compared to the anterior callus (*P* < 0.05 for each), while Tb.Sp exhibited no significant difference (Fig. 6d-e). In the OVX group, on the other hand, BMD of the posterior callus was significantly increased, and Tb.Sp was significantly decreased (*P* < 0.05 for each *vs.* anterior callus).

### TRAP staining

**Fig. 7:**
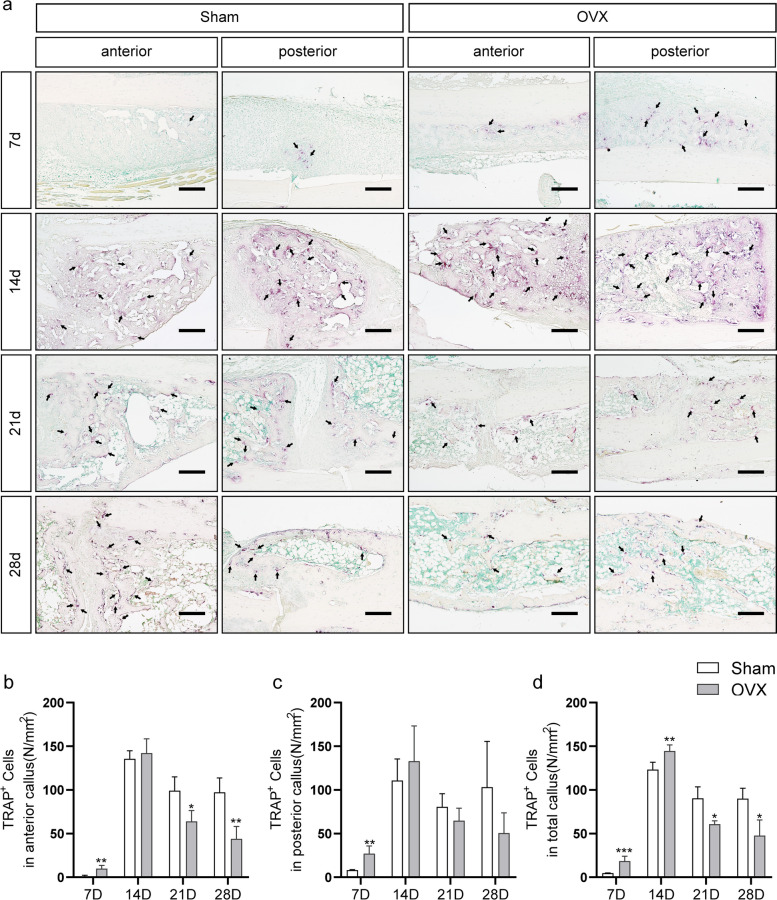
TRAP-stained sections with quantitation of osteoclast density (N/mm^2^) in anterior and posterior calluses. The black arrow presents osteoclast. Bar = 200 µm. Data are presented as the mean ± SD. ^*^P < 0.05 *vs.* sham group; ^**^P < 0.01 *vs.* sham group; ^***^P < 0.001 *vs.* sham group

**Fig. 8:**
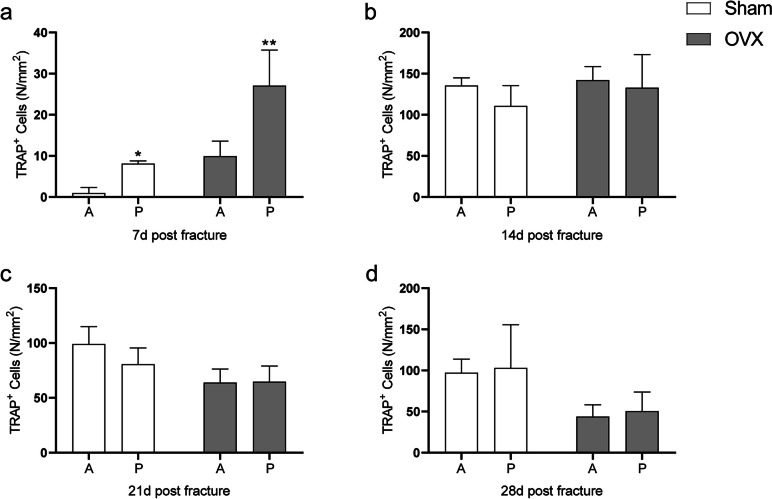
Analysis of TRAP stain in anterior and posterior callus. **a-d** Comparison of osteoclast density (N/mm^2^) between anterior and posterior calluses at 7, 14, 21, and 28 d post fracture. A: anterior callus; P: posterior callus. Data are presented as the mean ± SD. ^*^P < 0.05 *vs.* sham group; ^**^P < 0.01 *vs.* sham group; ^***^P < 0.001 *vs.* sham group

TRAP staining on serial histological sections was preformed to evaluate osteoclastogenesis on fracture healing (Fig. 7a). In the anterior callus, there were significantly more osteoclasts in the OVX group than in the sham group at 7 d post fracture, and significantly fewer than those in the sham group at 21 and 28 d post fracture (*P* < 0.05 for each; Fig. 7b). In the posterior callus, only at 7 days after fracture was osteoclast content significantly increased in the OVX group (*P* < 0.05), with there being no significant differences between the two groups at other time points (Fig. 7c). In the total callus, we found that osteoclasts were significantly increased in the OVX group at 7 and 14 d post fracture, while they were significantly decreased at 21 and 28 d after fracture (*P* < 0.05 for each; Fig. 7d). Higher osteoclast content was observed in the posterior callus of both sham and OVX groups at 7 d post fracture (Fig. 8a). No significant difference was found at the other three time points (Fig. 8b-d).

### Col-I expression

**Fig. 9:**
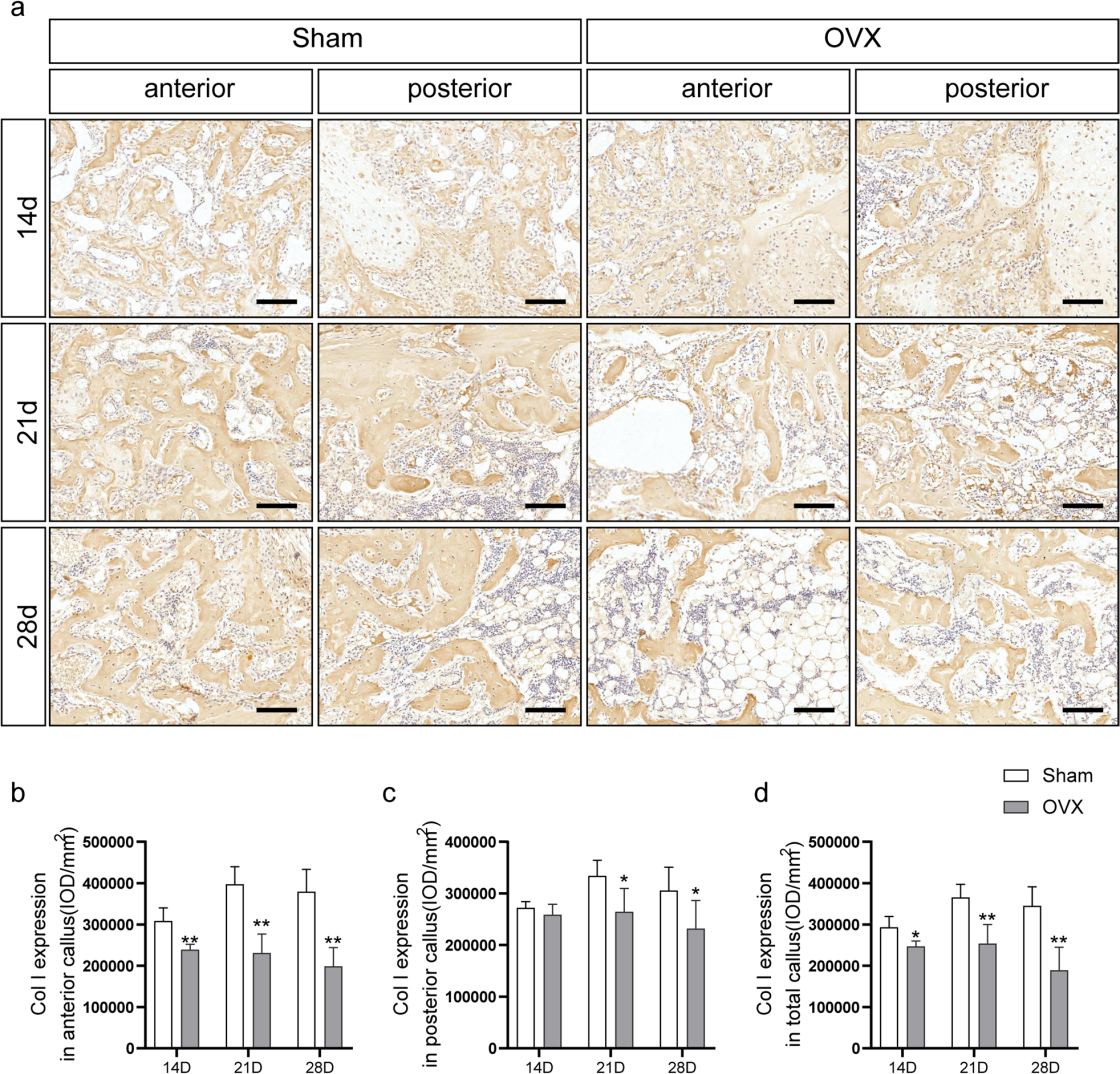
Immunochemistry of collagen-I. **a** Immunohistochemical staining for collagen-I in anterior and posterior calluses. **b-d** Quantitation of collagen-I expression in anterior, posterior, and total calluses. Bar = 100 µm. Data are presented as the mean ± SD. ^*^P < 0.05 *vs.* sham group; ^**^P < 0.01 *vs.* sham group; ^***^P < 0.001 *vs.* sham group

Type I collagen expression is shown in Fig. 9a. Quantitative results (Fig. 9b-d) showed that the expression of Col-I in the anterior and total callus showed the expression of Col-I in the OVX group was significantly lower than that in the sham group at 14, 21, and 28 d post fracture (*P* < 0.05 for each). In the posterior callus, the expression of Col-I decreased in the OVX group at 21 and 28 d post fracture (*P* < 0.05 for each), while, at 14 d post fracture, there were no significant differences between the two groups.

### Three-point bending test

**Fig. 10:**
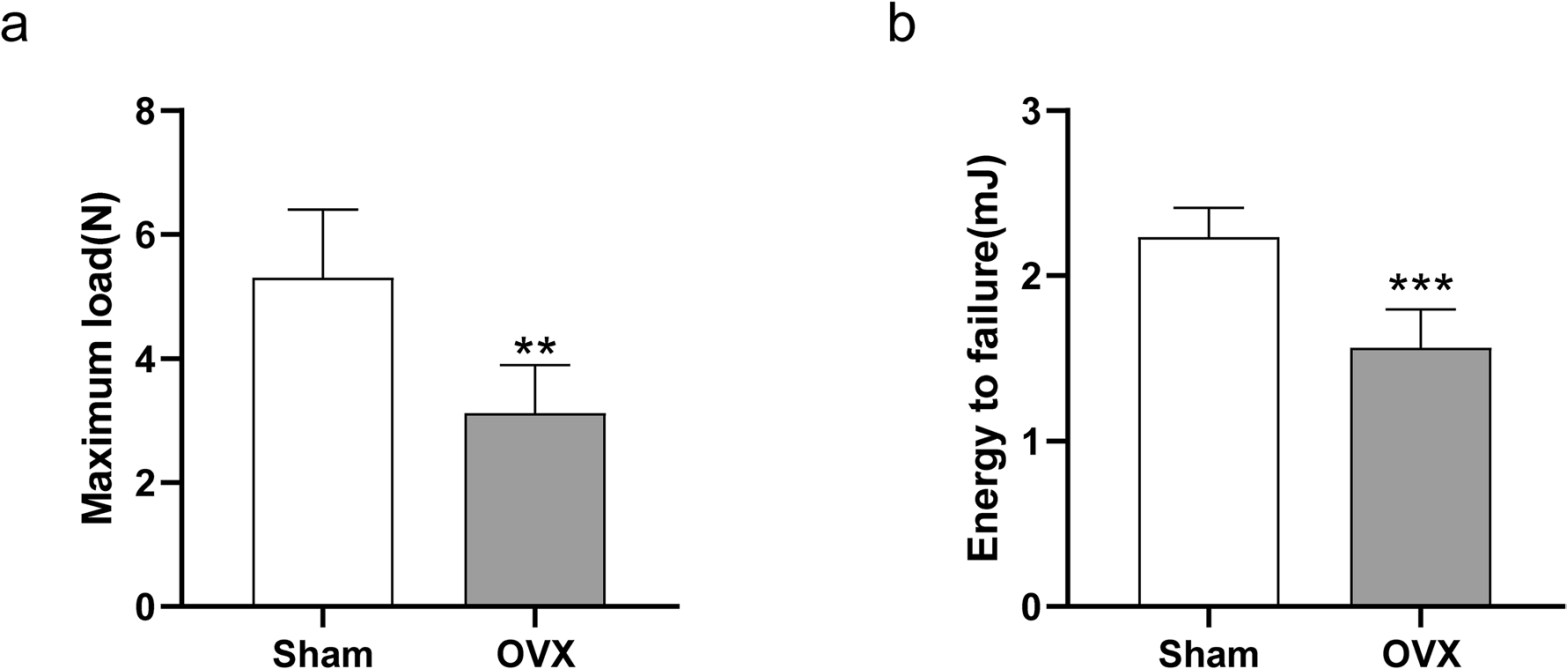
Biomechanical test. **a** Maximum load. **b** Energy to failure. Data are presented as the mean ± SD. ^*^P < 0.05 *vs.* sham group; ^**^P < 0.01 *vs.* sham group; ^***^P < 0.001 *vs.* sham group

The three-point bending test was used to detect the biomechanical properties of each callus using specimens at 28 d post fracture (Fig. 10). Compared with the sham group, the maximum load decreased by 41%, and the energy-to-failure decreased by 29% in the OVX group (all *P* < 0.05).

## Discussion

In this study, we have defined a new, closed, semi-fixed, distal tibial fracture model. In this model, the healing progress of the anterior and posterior callus in the sham group was inconsistent, with the posterior callus showing excessive bone resorption and lower bone mass phenotype compared with the anterior site. In contrast, at 28 days after fracture, the posterior callus was more mineralized than the anterior callus in the OVX group. Nevertheless, although the fracture healing of the sham group showed a special phenotype in this mode, the progress and quality of fracture healing were still better than those of the OVX group.

Cheap, easy to breed, convenient for gene editing, and short necessary bone healing time have made mice more widely used in experimental research on fracture healing [22–24]. Among them, the femur and the tibia are the two most commonly used modeling sites. The triangular configuration of the tibia and its long, curved axis are often considered less ideal than the femur, but the long axis of the bone in the middle and lower third of the tibia (below the tibiofibular joint) is not curved, and its length also facilitates biomechanical testing [25]. Compared with the femur, the distal tibia possesses a distinct advantage; the thin, soft tissue covering makes the tibia more prone to transverse fractures [26–28]. Previous studies have proven the importance of soft tissue adjacent to the fracture site [29]. The anatomical structure of the distal tibia of mice is similar to that of humans, which makes it possible to simulate the fracture of human distal tibias and study the mechanism(s) thereof. For these reasons, we chose the distal tibia to establish the closed fracture model.

Increasing mechanical load, such as during exercise or intense muscle contraction, is able to enhance bone mass accumulation and increase bone strength [30]. Mechanical stimulation is equally vital during fracture healing [31], and early weight bearing is an important part of fracture rehabilitation in clinical practice [32]. Appropriate mechanical stimulation at the right time after fracture can promote both bone and cartilage formation. A finite element analysis was performed on the mouse tibia. Under an axial load, the stress on the posterior side of the distal tibia was higher than that on the anterior side [33]. The 3D reconstructed fracture morphology compiled by Colucci et al*.* [34] also indicated that stress transfer at the injury site of the distal tibia fracture model was biased toward the posterior side. Congruent with these observations, when comparing the content of bony callus in anterior and posterior callus in the same group, we tended to observe either no difference or better healing on the posterior side than on the anterior side.

In our study, the fractures in the sham group showed a specific morphology at 28 days post fracture, with a dense anterior bony callus and a sparse posterior one. Comparison of the microstructural parameters of the anterior and posterior callus revealed that the BMD, BV/TV, and Tb.N of the posterior callus were significantly lower than those of the anterior callus, which was different from our previous supposition. Therefore, we performed sagittal sections of specimens at 7, 14, 21, and 28 d post fracture to compare the differences between the anterior and posterior calluses of the same specimens. Starting at 14 days after fracture, the posterior callus began to show significantly less bony callus content than the anterior site, and a significantly larger medullary cavity and more adipocytes appeared in the posterior site. A similar situation occurred in the samples at 28 d post fracture. It is clear that the bony callus in the posterior callus is excessively resorbed. This is also evidenced by the significantly higher content of TRAP-positive cells in the posterior callus of the sham group compared to the anterior site 7 days after fracture in this experiment.

The same mouse model has been used in some studies, but similar phenomena have not been reported. Xin Li et al*.* [15] use 30G needles as fixation pins to fix a tibia fracture in 8-week-old male mice. The younger age (2 months old) of the mice and the non-standardized fracture position just around the tibiofibular junction may result in the appearance of this different phenotype. Studies by Oranger et al*.* [35] and Hiltunen et al*.* [36] also investigated the fracture healing in distal tibia. In contrast to our study, 0.2–0.22 mm diameter 316 stainless steel fixation pins were used to fix the fracture bone. In way of a potential explanation for this discrepancy, we found that we used a thicker intramedullary pin, 0.3 mm diameter 06Cr19Ni10 stainless steel. While the thicker intramedullary pin provided strong fixation and maintained bone morphology, in combination with the direction of stress in the distal tibia, the transmission of mechanical loads in the posterior tibia may have been compromised. This may have resulted in a stress shielding phenomenon similar to that seen in clinical work, which occurs around the insertion site of bone tissue implants. This can alter the physiological load of the callus and thus cause bone resorption [37].

Interestingly, the same trend was not observed for calluses in the OVX group. Even after 28 days post fracture, the BMDs of the calluses were significantly higher on the posterior side of the OVX group than on the anterior side. Histology showed the same trend, with a significantly higher proportion of bony callus on the posterior side than on the anterior side 28 days after fracture. Similar results were described by Hiltunen et al*.* [36]. Whole-body low-magnitude high-frequency vibration (LMHFV) promoted fracture healing in OVX mice, but it had no or opposite effects in healthy mice. This observation suggests that the response of the bony callus to stress stimulation is more sensitive in estrogen-depleted mice than in normal mice [10] and supports our findings that there were no significant differences in the phenotypes of the anterior and posterior calluses in the OVX group. This indicates that, even though the fixed pin may cause abnormally reduced stress, it can still stimulate bone repair without a site-specific inconsistent manner in estrogen deficiency mice.

Low circulating estrogen or estrogen depletion reduces the adaptive response of bone to mechanical loading [38]. Recent study has shown that estrogen deficiency alters the in situ expression of key Ca^2+^ signaling in osteocyte processes, resulting in dysregulation of the osteocyte mechanosome complex. Osteocytes lose the ability to sense small mechanical stimuli, and mechanosensation is reduced [38]. In addition, the perception of mechanical stimuli by osteoblasts is highly dependent on gap junction-mediated intercellular communication [39]. This may explain the absence of a stress shielding-like phenomenon in the OVX group. However, an increase in osteoclasts in the posterior callus was also observed in the OVX group 7 d post fracture. Thus, we considered that another feature of estrogen deficiency – that of systemic high bone turnover – could also be responsible. In the total bony callus, we observed a significantly higher content of osteoclasts at 7 and 14 d post fracture compared to the sham group. This likely resulted in the woven bone of anterior callus also being absorbed by active osteoclasts, leaving no significant difference in bony callus between the anterior and posterior calluses.

Bone integrity is disrupted after a fracture, and the load that should be transmitted by the posterior lateral tibial cortical bone is instead absorbed by the intramedullary pin. We observed by histology that, at 28 d post fracture, a better bridging connection had formed at the posterior callus in the OVX group. We hypothesize that the decreased sensitivity of the bone to mechanical stimuli due to estrogen deficiency reduced the excessive resorption of the posterior callus. As a result, fracture healing was delayed, but the integrity of the posterior side of the distal tibial fracture was restored. Thus, the rise in stress transmitted via the posterior tibial callus promoted bone formation.

**Fig. 11:**
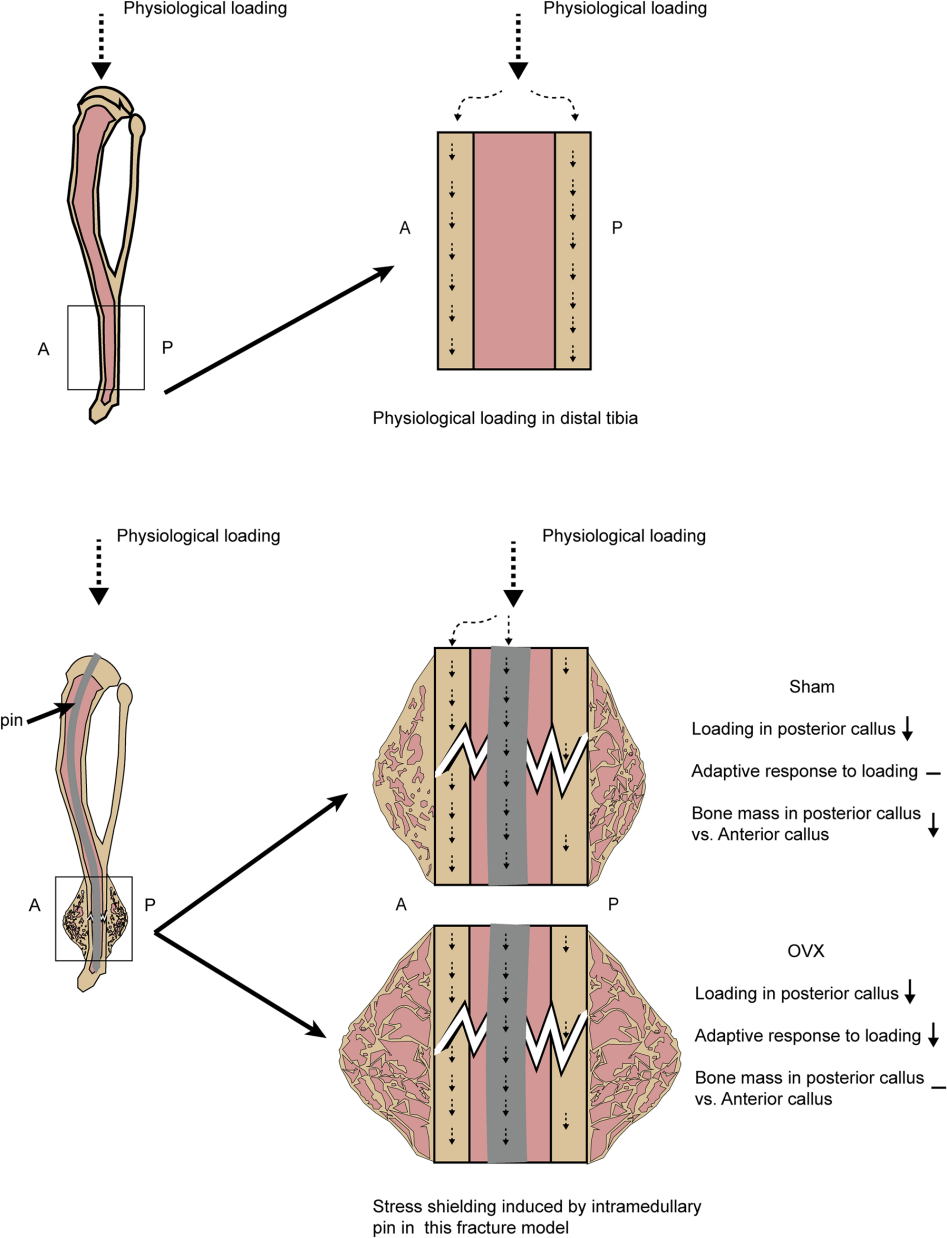
Stress transmission in semi-fixation distal tibia fracture model

Delayed healing and impaired bony callus quality in OVX-induced osteoporotic fractures are associated with delayed expression of estrogen receptors [40]. The peak expression of estrogen receptors in the bony callus of OVX-induced osteoporotic fractures was delayed compared to normal fractures. However, stress stimulation in the absence of estrogen promoted the expression of estrogen receptor α and thus increased bone formation [9, 10]. These may explain the higher bone volume of the posterior scab in the OVX group compared to the anterior side 28 d post fracture (Fig. 11). However, weight changes also should be taken into consideration. Previous studies showed that the body weight of OVX mice was significantly higher than that of the control group at 8 weeks postoperatively [41–43]. In our model, this increased weight may have helped balance the uneven stress transmission caused by the fixation pins.

The effects of OVX-induced osteoporosis on fracture healing have been well studied. Healing of osteoporotic fractures is impaired at both early and late stages [9, 44], exhibiting delayed fracture bridging and bony callus maturation, disturbed immune response, and reduced angiogenesis, similar to that observed in osteoporotic patients [45–47]. Thus, the delayed resorption of cartilage callus, poorer bone quality, and lower biomechanical performance relative to the sham group observed in our study align with previous research findings.

These results characterize the inconsistent healing process between anterior and posterior callus during distal tibial fracture healing, but do not fully elucidate the molecular mechanisms affecting healing in this mouse model. Our model does not allow us to obtain the biomechanical data of anterior and posterior calluses for comparison. However, this is the first time that the effect of OVX-induced osteoporosis on fracture healing of the distal tibia fracture has been directly measured. While previous studies have generally used large animals such as dogs to investigate the stress shielding effect [13], our model proposes new possibilities for future applications of mouse fracture models.

## Conclusion

In this study, a mouse model of a semi-fixed distal tibial closed fracture was successfully established. Excessive bone resorption of the posterior callus in this model impaired normal fracture healing, but not in OVX-induced osteoporotic bone. Although the stress shielding effect was not observed in OVX group, the bone healing impairment caused by OVX still existed. This investigation suggests that this fracture model may hold potential for studies on distal tibial fractures and stress shielding.

## Data Availability

The datasets used and/or analysed during the current study are available from the corresponding author on reasonable request.

## Abbreviations

OVX: Ovariectomy
TRAP: Tartrate-resistant acid phosphatase
Col-I: Type 1 collagen
BMD: Bone mineral density
BV/TV: Bone volume/total volume
Tb.N: Trabecular number
Tb.Sp: Trabecular separation
